# Distinct Identity of GLP-1R, GLP-2R, and GIPR Expressing Cells and Signaling Circuits Within the Gastrointestinal Tract

**DOI:** 10.3389/fcell.2021.703966

**Published:** 2021-09-29

**Authors:** Nadya M. Morrow, Antonio A. Hanson, Erin E. Mulvihill

**Affiliations:** ^1^Energy Substrate Laboratory, University of Ottawa Heart Institute, Ottawa, ON, Canada; ^2^Department of Biochemistry, Microbiology and Immunology, University of Ottawa, Ottawa, ON, Canada; ^3^Montreal Diabetes Research Center CRCHUM-Pavillion R, Montreal, QC, Canada; ^4^Centre for Infection, Immunity and Inflammation, University of Ottawa, Ottawa, ON, Canada

**Keywords:** glucagon-like peptides, intestine, incretins, metabolism, glucose-dependent insulinotropic polypeptide

## Abstract

Enteroendocrine cells directly integrate signals of nutrient content within the gut lumen with distant hormonal responses and nutrient disposal via the production and secretion of peptides, including glucose-dependent insulinotropic polypeptide (GIP), glucagon-like peptide 1 (GLP-1) and glucagon-like peptide 2 (GLP-2). Given their direct and indirect control of post-prandial nutrient uptake and demonstrated translational relevance for the treatment of type 2 diabetes, malabsorption and cardiometabolic disease, there is significant interest in the locally engaged circuits mediating these metabolic effects. Although several specific populations of cells in the intestine have been identified to express endocrine receptors, including intraepithelial lymphocytes (IELs) and αβ and γδ T-cells (*Glp1r*+) and smooth muscle cells (*Glp2r*+), the definitive cellular localization and co-expression, particularly in regards to the *Gipr* remain elusive. Here we review the current state of the literature and evaluate the identity of *Glp1r, Glp2r*, and *Gipr* expressing cells within preclinical and clinical models. Further elaboration of our understanding of the initiating G-protein coupled receptor (GPCR) circuits engaged locally within the intestine and how they become altered with high-fat diet feeding can offer insight into the dysregulation observed in obesity and diabetes.

## Introduction

Extending between the stomach and the colon lies among the most significant hormone-producing and immunological organs responsible for nutrient digestion and absorption: the small intestine. Within the small intestine lumen, the surface area is ideally maximized to enhance nutrient absorption through villi and microvilli, which increase intestinal surface area by 30–600-fold (Kiela and Ghishan, 2016). A single layer of epithelial cells lines the surface of each villus to serve as the gateway for controlled nutrient absorption and a barrier to dietary antigens and diverse microorganisms (Turner, 2009). Absorptive enterocytes populate the villus tip and account for >80% of intestinal epithelial cells. The remaining mature cell types include mucin-producing goblet cells, antimicrobial defensins-producing Paneth cells, peptide-hormone-producing enteroendocrine cells, and cytokine-producing tuft cells which reside throughout the epithelium (Ensari and Marsh, 2018). Shallow invaginations surrounding each villus are intestinal crypts and the site of cell division: highly mitotic stem cells that give rise to progenitor cells, which in turn proliferate to become mature epithelial cells (Gehart and Clevers, 2019). The continuous supply of progenitor and new epithelial cells physically promotes the transit of the latter from the crypts up to the villus tip, where they populate the newly vacant area of previously shed apoptotic epithelial cells (Gehart and Clevers, 2019). Therefore, in addition to the maximized absorptive surface area, the constantly renewing barrier protects the internal environment from the harsh conditions of the intestinal lumen. This single epithelial layer sits on a basement membrane surrounding a connective tissue core called the lamina propria, which contains lymphocytes and innate immune cells (Ensari and Marsh, 2018). Each villus is supplied by an arteriole that forms a capillary network, a venule that drains into larger vessels at the crypts (Ensari and Marsh, 2018), and 1–2 lacteals, which are terminal lymphatic vessels of the mesenteric network. Pericytes coat villus blood vessels while smooth muscle cells coat lacteals. The lamina propria also contains connective tissue scaffolds, enteric nerves, fibroblasts, and smooth muscle cells (Bernier-Latmani and Petrova, 2017). The lamina propria is encapsulated by a myofibroblast shell that directly contacts the vascular network. From the villus tip to the muscularis mucosa, onto which villi are anchored, is considered the mucosal layer. The submucosal layer contains blood and lymphatic vessels and a plexus of parasympathetic nerves (Bernier-Latmani and Petrova, 2017), while the smooth muscle cell-rich muscularis propria allows for contractile peristalsis (Collins et al., 2021). The final outer layer of the intestine is the serosa, composed of loose connective tissue and squamous epithelial cells (Collins et al., 2021), which is continuous with the mesentery. The mesentery supports the intestine in the peritoneum and also contains blood vessels, nerves, and lymphatics (Argikar and Argikar, 2018). The coordination of barrier function with nutrient absorption and transit is governed by a complex integration of signals, including local enteroendocrine production of peptide hormones, which impacts both the dynamic and highly efficient process of nutrient assimilation.

In addition to its expression in the pancreas, proglucagon is also produced in enteroendocrine L cells throughout the small and large intestine (Jorsal et al., 2018). Here, posttranslational processing of the 160 amino acid proglucagon by prohormone convertase 1/3 (PC1/3) yields active peptides glicentin, glucagon-like peptide 1 (GLP-1), intervening peptide 2 (IP2), and glucagon-like peptide 2 (GLP-2) (Mojsov et al., 1986; Orskov et al., 1986). Evidence for gut-derived glucagon is observed in patients with a total pancreatectomy during a glucose tolerance test (Lund et al., 2016). GLP-1, first identified from amino acids 1–37 and 1–33 (Drucker et al., 1986), is active upon N-terminal truncation, where GLP-1(7–37) and GLP-1(7–36)amide are physiologically active with well-defined roles in promoting nutrient-stimulated insulin secretion (Drucker et al., 1987; Holst et al., 1987). The active form of GLP-2 in tissue and circulation is the complete 1–33 amino acid (Brubaker et al., 1997) upon C-terminal truncation of 2 amino acids (Orskov et al., 1989b) with a well-defined role of acting locally to promote nutrient uptake, barrier function and gut growth.

Glucose-dependent insulinotropic polypeptide (GIP) is a peptide hormone expressed and secreted by intestinal K enteroendocrine cells. GIP is derived from a 144 amino acid (rodent) (Higashimoto et al., 1992; Higashimoto and Liddle, 1993; Tseng et al., 1993) or 153 amino acid (human) (Takeda et al., 1987) precursor, proGIP. Most K cells express PC1/3, which cleaves proGIP at Arg65, resulting in the biologically active GIP(1–42) (Ugleholdt et al., 2006) and stored in secretory granules (∼450 nm) (Buchan et al., 1978). A small population of K cells express PC2 instead of PC1/3, resulting in GIP(1–31), which is amidated by peptidyl-glycine α-amidating monooxygenase, resulting in GIP(1–30) (Fujita et al., 2010). Initially discovered in 1973 for its role in inhibiting gastric acid secretion in excised canine stomach pouches, and later shown to not have this effect in humans (Meier et al., 2004a), GIP promotes nutrient-stimulated insulin secretion and increases glucagon secretion in the fasted state but not in patients with type 2 diabetes (Baggio and Drucker, 2007; Christensen et al., 2011).

The physiological concentrations of the peptide hormones GIP, GLP-1, and GLP-2, are tightly controlled by the nutrient-sensing abilities of their respective enteroendocrine cells. Additionally, the serine protease dipeptidyl peptidase 4 (DPP4) limits the bioavailability of GIP, GLP-1, and GLP-2 by cleaving the first two amino acids, rendering them inactive (Deacon et al., 1995a; Knudsen and Pridal, 1996; Hansen et al., 1999). In healthy humans, GIP has a circulating half-life of 7 min (Meier et al., 2004b), GLP-1 has a circulating half-life of 1–2 min (Deacon et al., 1995b), and GLP-2 has a circulating half-life of 7 min (Drucker et al., 1997; Hartmann et al., 2000). GIP concentrations are much greater than GLP-1 in the postprandial state (Meek et al., 2021). Prolonged activation of GLP-1, GLP-2, and GIP receptors is achieved through receptor agonists resistant to DPP4 cleavage or through compounds that inhibit DPP4 activity (Jeppesen et al., 2005; Baggio and Drucker, 2007).

This review highlights the biology and paracrine roles of GLP-1, GIP, and GLP-2 in integrating the response to food intake with the maintenance of the structure and function of the gut as it relates to nutrient absorption. We critically assess experiments reporting the identification and role(s) of GPCRs: GIP receptor (GIPR), GLP-1 receptor (GLP-1R) and GLP-2 receptor (GLP-2R) in intestinal physiology. We also emphasize both preclinical and clinical studies identifying how agonists to these receptors transduce their metabolic actions. We limit our discussion to intestinal biology and the resulting metabolic phenotypes and refer readers interested in other aspects of the GLP-1R, GLP-2R and GIPR biology to access other excellent publications (Campbell, 2021; Ghislain and Poitout, 2021; Gribble and Reimann, 2021; McLean et al., 2021).

## Gut Hormonal Responses to Nutrients

Enteroendocrine cells are highly sensitized to nutrient intake due to their polarized shape, direct contact with the lumen, and proximity to the vasculature for peptide secretion. Upon ligand-receptor binding and depolarization, hormone-containing granules fuse with the lateral and basal membrane for discharge into the villus capillaries (Paternoster and Falasca, 2018). This idealistic design favors rapid and precise peptide delivery in circulation to initiate signaling through their respective receptors to control metabolism. Additionally, enteroendocrine cells are equipped with GPCRs and transporters to sense the macronutrients and release the appropriate hormones (Spreckley and Murphy, 2015). These include: G-protein coupled receptor (GPCR) family C group 6 subtype A (GPRC6A), Taste Rs (amino acids), G-protein coupled receptor 93 (GPR93) (peptones), free-fatty acid receptor 2 (FFAR2), free-fatty acid receptor 3 (FFAR3), short-chain fatty acid (SCFA), free-fatty acid receptor 1 (FFAR1), free-fatty acid receptor 4 (FFAR4), long-chain fatty acid (LCFA) and G-protein coupled receptor 119 (GPR119) [oleoylethanolamide (oea)] are some of the macronutrient-sensing receptors present on enteroendocrine cells (Spreckley and Murphy, 2015). First, we begin with an overview of the regulation of the synthesis, secretion, and location of these peptides.

### GIP Expression and Secretion

**FIGURE 1:**
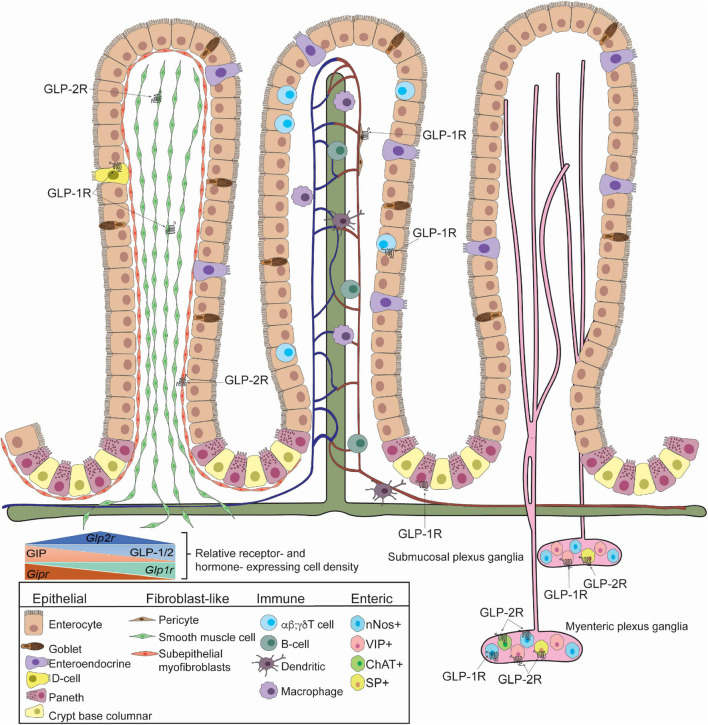
GLP-1R and GLP-2R-expressing cells in the small intestine identified in mice. Villus architecture is organized as fibroblast-like cells **(Left)**, blood, lymph and immune cells **(Middle)**, enteric cells **(Right)**. GLP-1R is expressed in somatostatin-secreting enteroendocrine cells, smooth muscle cells, pericytes, Paneth cells, Tαβ cells, Tγδ cells, submucosal and myenteric neurons. GLP-1R is detected in neuronal nitric oxide (nNOS)+ neurons. GLP-2R is detected in smooth muscle cells, subepithelial myofibroblasts, submucosal and myenteric neurons. Specifically, GLP-2R is expressed in nNOS+ cells, vasoactive intestinal polypeptide (VIP)+ cells, choline-acetyltransferase (ChAT)+ cells, and substance P (SP)+ cells. The relative receptor- and hormone- expression cell density within the small intestine is depicted (inset). Studies highlighted here did not determine co-expression of GLP-1R and GLP-2R and specific identity of GIPR-expressing cells is unclear.

GIP mRNA (Tseng et al., 1993) and concentration (Bryant et al., 1983) are enriched in duodenal and jejunal mucosal tissues in rodents and humans compared to the distal ileum (Figure 1). Forty-eight hours of fasting in rats significantly decreases *Gip* mRNA (∼44%) in the proximal small intestine compared to rats maintained on a chow diet. At the same time, GIP peptide concentrations do not change with fasting or feeding (Higashimoto et al., 1995), suggesting that synthesis and secretion are relatively synchronized. K-cells in the proximal small intestine contain more GIP protein and secrete more GIP in response to intestinal lard oil perfusion than distal K cells (Iwasaki et al., 2015). *GIP* expression is significantly greater in both the small intestine and colon of patients with Type 2 diabetes than healthy individuals (Jorsal et al., 2018). Interestingly, in patients with type 2 diabetes, the density of PC1/3-positive cells decreases while both the expression and density of PC-2 positive cells increases (Jorsal et al., 2018). Nutrient stimulation of GIP secretion has also been reviewed here (Pais et al., 2016; Reimann et al., 2020).

In K cells, regulatory factor X6 (Rfx6) is a transcription factor that binds to the *Gip* promoter to increase *Gip* mRNA expression (Suzuki et al., 2013). Intestine-specific gene transfer experiments of pancreatic and duodenal homeobox-1 (*Pdx1*) siRNA in 8–10-week-old mice reveal that posteriori suppression of Pdx1 decreases K-cell number, intestinal GIP protein and mRNA expression, and GIP secretion in response to an oral glucose tolerance test (OGTT) (Ikeguchi et al., 2018). The number of K cells and their *Gip* mRNA content increases with age, which corresponds to the GIP hypersecretion observed in 1 year old mice compared to 3–4-month-old mice (Ikeguchi et al., 2018). Moreover, transcription factor *Pdx1*, but not *Rfx6* mRNA increases with age in K cells (Ikeguchi et al., 2018). Both dietary fat and carbohydrate stimulate GIP secretion (Pederson et al., 1975; Brown and Otte, 1979; McCullough et al., 1983). Intraduodenal perfusion of 20% Lipomul significantly increases duodenal *Gip* mRNA at 30 and 60 min compared to saline control (Tseng et al., 1993). Both glucose (4-fold) and fat (2.5-fold) ingestion increase *Gip* mRNA expression compared to chow-diet feeding (Higashimoto et al., 1995). High-fat feeding does not increase K-cell number in mice, instead, it increases GIP protein content and mRNA expression, which correlates to increased *Rfx6* and *Pdx1* mRNA expression (Suzuki et al., 2013). Therefore, through different mechanisms, both diet-induced obesity and aging act on the gut to increase GIP reserves for secretion into circulation.

#### Nutrient Stimulated GIP Secretion

GIP secretion increases more rapidly in response to simple, fast-absorbing carbohydrates compared to complex, slow-absorbing carbohydrates (Collier et al., 1984). Plasma GIP levels rise significantly higher upon oral fat consumption compared to glucose in mice (Shibue et al., 2015) and in humans (Yamane et al., 2012). Further, ingestion of a mixed carbohydrate and fat meal significantly increases plasma GIP levels compared to carbohydrates alone in healthy humans (Collier et al., 1984) but this increase is not as great as ingestion of fat alone in healthy humans (Creutzfeldt et al., 1978). GIP secretion in response to oral fat is greater in patients with obesity and glucose intolerance, and does not change with the addition of glucose to the meal (Creutzfeldt et al., 1978).

GIP concentrations in the bloodstream are the highest in hepatic portal plasma, however, lymph GIP concentrations are ∼3-fold higher upon the same stimulus (D’Alessio et al., 2007; Lu et al., 2008), indicating peptide transit from K cells to villus lacteals. Intraduodenal delivery of a bolus of dextrin and a bolus of Liposyn (20%) in rats each induce ∼800 and ∼400 pg/mL peaks, respectively, in lymph GIP concentrations at 60 min (Lu et al., 2008). However, the peak secretion rate occurs at 30 min for Liposyn (1,159 ± 393 pg/h) and at 60 min for dextrin (2,410 ± 566 pg/h). The combination of dextrin and Liposyn delivery significantly increases GIP secretion at 30 min (2,094 ± 241 pg/h) and at 60 min (8,027 ± 1,057 pg/h) compared to saline, dextrose alone, and Liposyn alone (Lu et al., 2008). These data suggest that glucose and lipids stimulate K cells differently, therefore potentiating release when administered together. Indeed, preventing micelle formation via common bile duct ligation abolishes GIP secretion upon a lard gavage compared to sham controls, independent of meal transit (Shibue et al., 2015). As dietary fatty acids are assembled into lipoproteins in intestinal enterocytes for subsequent circulatory transport, blocking lipoprotein transit from endoplasmic reticulum (ER) to Golgi by Pluronic L-81 in rats robustly reduces (∼4.5-fold) lymph GIP levels and secretion rates in response to Liposyn to levels similar to saline controls (Lu et al., 2012). Therefore, GIP secretion from K cells in response to Liposyn requires post-Golgi chylomicron transit in enterocytes, not lipid absorption alone (Lu et al., 2012). GIP secretion increases in response to chylomicrons alone and the presence of glucose in both murine and human duodenal cultures in a dose-dependent fashion (Psichas et al., 2017). Glucose stimulation of chylomicron secretion is well documented (Robertson et al., 2003; Stahel et al., 2019; Xiao et al., 2019) where glucose promotes chylomicron secretion from lipid stores in enterocytes (Stahel et al., 2019), which may provide additional stimulus for GIP secretion. Co-intraduodenal infusion of mixed nutrients (carbohydrate, dextrose) and lipid (20% Liposyn) in rats significantly increase GIP secretion in lymph to a greater extent than either nutrient at the same meal caloric value alone, suggesting a synergistic effect (Lu et al., 2008). Consistent with glucose-stimulated chylomicron secretion, lymph TG values are the same when Liposyn accounts for half of the meal calories (the other half being dextrose) compared to a full Liposyn meal (Lu et al., 2008). Experiments measuring glucose-stimulated GIP secretion after inhibiting chylomicron release (Pluronic 8–18) or basolateral hydrolysis of chylomicrons (poloxamer-407) may help delineate the exact contribution of each nutrient. Nevertheless, the requirement of chylomicron formation for GIP secretion from proximal K cells corresponds to a location-specific stimulus. Taken together, these studies demonstrate the complex integration of pathways governing GIP secretion and intestinal lipid metabolism.

**FIGURE 2:**
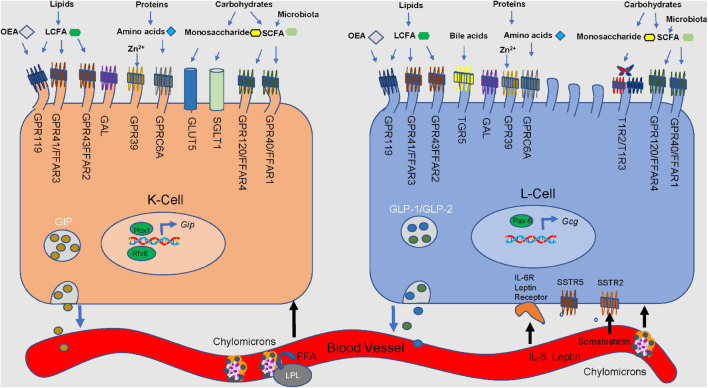
Nutrient stimulation of GIP from K cells and GLP-1 and GLP-2 from L cells. Microvilli protrude from the apical side of the cell, extending toward the lumen. Incoming macronutrients from the lumen are detected and sensed by receptors present on the apical border. Carbohydrates are divided into monosaccharides and short-chain fatty-acids (SCFA). Glucose is transported through the sodium-glucose linked transporter (SGLT1). Glucose passes through the glucose transporter 2 (GLUT2) present on the basolateral membrane, and into the bloodstream. GLUT5 is responsible for sensing fructose. Present on only L cells, sweet receptors (T1R2/T1R3) also sense glucose. Short-chain fatty acids (SCFA) are derived from microbial fermentation, and are sensed by the free fatty acid receptors (GPR40/FFAR1 and GPR120/FFAR4). Long-chain fatty-acids are sensed by GPR43/FFAR2 and GPR41/FFAR3, along with G-protein coupled receptor 119 (GPR119). GPR119 is also a receptor for oleoylethanolamide (OEA). Amino acids are detected by GPR9C6A. GPR39 and Takeda G-protein coupled receptor 5 (TGR5) detect luminal zinc and bile acids, respectively. Galanin receptor (GAL) acts to inhibit both GLP-1 and GIP secretion. Lipoprotein lipase is involved in the production of LCFA and monoacylglycerols. Somatostatin receptors 2 and 5 (SSTR2&5) sense somatostatin from D cells. Regulatory factors x6 (Rfx6) and insulin promoter factor 1 (Pdx1) influence GIP expression and secretion.

The free fatty acid receptor GPR120 is enriched in proximal K cells while GPR40, GPR41, and GPR43 are significantly enriched in distal K cells (Iwasaki et al., 2015). GIP secretion is unaffected by FFA1 agonism (Am-1638) or antagonism (GW1100) in primary murine duodenal cultures (Psichas et al., 2017). GIP concentration in plasma over 120 min decreases by 75% in *Gpr120^–/–^* mice upon lard oil gavage compared to wild-type mice (Iwasaki et al., 2015). Correspondingly, intestinal perfusion experiments in *Gpr120^–/–^* mice reveal that GIP secretion is significantly reduced from both proximal and distal regions of the small intestine compared to wild-type controls (Iwasaki et al., 2015). Similarly, oral pretreatment with a GPR120 partial antagonist, grifolic acid methyl ether, reduces GIP secretion by 80% in response to lard oil gavage (Iwasaki et al., 2015). All GIP+ cells express fatty acid-binding protein 5 (FABP5) (Shibue et al., 2015). While whole-body elimination of FABP5 in mice does not impact GIP content or K cell number, these mice secrete significantly less GIP into plasma 60 min after a lard gavage compared to wild-type controls (Shibue et al., 2015). *Ex vivo* duodenal segments from *Fabp5*^–/–^ mice secrete significantly less GIP in response to oleic acid with 4 v/v% bile in media than tissues isolated from wild-type mice (Shibue et al., 2015). These data suggest that micelle-facilitated fatty acid uptake via FABP5 in response to luminal lipids significantly contributes to meal-stimulated GIP secretion (Shibue et al., 2015) (Figure 2).

Glucose stimulates GIP secretion only when administered orally, therefore requiring apical exposure to K cells. Curiously, intraduodenal infusion of glucose in healthy men does not significantly increase plasma GIP levels from baseline (Herrmann et al., 1995), suggesting a transit time dependency for glucose-stimulated GIP secretion. Glucose injection in the upper intestine significantly increases plasma GIP levels while glucose injection in the colon does not (Moriya et al., 2009). Perfusion of glucose, sucrose, galactose, maltose, 3-*O*-methylglucose, and *a*- or *B*-methylglucoside significantly stimulate GIP secretion, while mannose, 6-deoxygalactose, 2-deoxyglucose, myoinositol, fructose or lactose do not (Sykes et al., 1980). Therefore, active transport by the sodium-dependent hexose pathway is required for GIP secretion (Sykes et al., 1980). Indeed, sodium glucose co-transporter 1 (SGLT1) receptor is expressed only on the apical side of K cells and oral gavage of SGLT1 substrate, a-methyl-D-glucopyranoside, stimulates GIP secretion (Moriya et al., 2009). The necessity for apical glucose transport is demonstrated in *Sglt1^–/–^* mice, where glucose-stimulated GIP secretion is eliminated and levels rise only to the same extent as observed in the saline control (Gorboulev et al., 2012). Genetic elimination of K_ATP_ channels (*Kir6.2^–/–^* mice) significantly increases glucose absorption and glucose-stimulated GIP secretion, through a compensatory increase in duodenal *Sglt1* mRNA expression (Ogata et al., 2014). Preventing glucose absorption with phloridizin abolishes glucose-stimulated GIP secretion in healthy wild-type (Sykes et al., 1980) and *Kir6.2^–/–^* mice (Ogata et al., 2014), even in the presence of *a*-methyl-D-glucopyranoside (Moriya et al., 2009). Similar to humans, mice and rats with diabetes secrete more GIP in response to oral glucose. Fructose transporter, GLUT5, is expressed on K-cells; however, fructose does not stimulate GIP secretion in healthy humans, rats, or mice (Kuhre et al., 2014; Seino et al., 2015). Fructose significantly increases GIP secretion in streptozotocin-treated, hyperglycemic mice in a K_ATP_-dependent manner (Seino et al., 2015) and in *ob/ob* mice (Flatt et al., 1989). This is further supported by the inability of phlorizin to prevent glucose-induced GIP secretion in streptozotocin-treated, hyperglycemic mice, where complete blockage of GIP secretion is only achieved in these mice upon both phlorizin and K_ATP_ channel activation (diazoxide) (Ogata et al., 2014).

Non-nutrient promoters of GIP secretion include oral administration of ZnCl_2_ to non-fasted mice, which increases GIP secretion 26% via K cell expression of GPR39 (Moran et al., 2019). Additionally, associated metabolic improvements with ZnCl_2_ administration are lost in *Gipr^–/–^* mice (Moran et al., 2019). Galinin is a centrally and peripherally synthesized neuropeptide and its receptor (GAL_1_) is expressed in K cells (Psichas et al., 2016). Both galinin and GAL_1_ agonist (M617) significantly inhibit IBMX−stimulated GIP secretion from primary duodenal cultures (Psichas et al., 2016). Oral administration of progesterone significantly increases glucose-stimulated GIP secretion (5 min) in male wild-type and *Glp1r^–/–^Gipr^–/–^* (double incretin receptor knockout; DIRKO) mice, but not in *Glp1r^–/–^* mice (Flock et al., 2013) (Figure 2).

#### GIP Secretion and the Microbiome

Glucose-dependent insulinotropic polypeptide levels are increased with subtherapeutic antibiotic therapy (STAT) (Cho et al., 2012) while other hormones are unaffected. It is suspected that levels are greater due to the increased abundance of Firmicutes and subsequent SCFA production (Martin et al., 2019), however, further studies to confirm this hypothesis are required.

### Expression and Secretion of GLP-1 and GLP-2

GLP-1+ cells reside in crypts and the villus epithelium; their density increases distally with the highest abundance in the ileum in rodents (Figure 1) and the colon in humans (Eissele et al., 1992). Within L cells, GLP-1 is stored in granules (Eissele et al., 1992) in its active form (7–36 amide) in the small (Orskov et al., 1989a) and large intestine (Deacon et al., 1995b). Forty-eight hours of fasting in rats significantly reduces ileal *Gcg* mRNA (25–50%), which was associated with a 41–60% decrease in plasma bioactive GLP-2 (Nelson et al., 2008). Both plasma GLP-2 and ileal *Gcg* mRNA levels were restored upon 2 days of refeeding or 4 days of continuous intragastric, but not intravenous, refeeding with total parenteral nutrition (TPN) solution (32% energy from fat 68% energy from dextrose) (Nelson et al., 2008). Colonic L cells contain twice as much GLP-1 peptide than proximal intestine L cells (Reimann et al., 2008). Both colonic *GCG* expression and GLP-1+ cell density increase in patients with type 2 diabetes compared to healthy individuals (Jorsal et al., 2018). By contrast, while *PCSK1/3* mRNA increases in patients with diabetes compared to healthy individuals, the density of PC1/3-positive cells decreases (Jorsal et al., 2018), suggesting a posttranslational impact on GLP-1 availability. GLP-1+ cells are also found in the stomach fundus where concentrations are higher than in the antrum in both diet-induced obese rats and humans with obesity (Ribeiro-Parenti et al., 2021). In diet-induced obese mice, IBMX-stimulated GLP-1 release *ex vivo* is completely abrogated in the antrum (Ribeiro-Parenti et al., 2021). Interestingly, the remodeling of the gastric mucosa following Roux-en-Y gastric bypass (RYGB) bariatric surgery in humans is accompanied by a ∼2-fold increase in fundic GLP-1 positive cells; this increase is not observed in patients following vertical sleeve gastrectomy (VSG) surgery (Ribeiro-Parenti et al., 2021). This increase in fundic mucosal GLP-1 following RYGB but not VSG was consistent in diet-induced obese rats, where instead, VSG surgery induced a 50% increase in GLP-1+ cells in the antrum (Ribeiro-Parenti et al., 2021). This increase was associated with a 1.5-fold increase in portal plasma GLP-1 upon gastric glucose stimulation in diet-induced obese VSG rats compared to diet-induced obese sham controls, suggesting that antral GLP-1 producing cells contribute significantly to portal GLP-1 (Ribeiro-Parenti et al., 2021). However, further experiments preventing GLP-1 secretion from ileal L cells will be required to precisely assess the contribution from the stomach after surgery.

#### Nutrient Stimulated GLP-1 Secretion

In healthy men, oral ingestion of corn oil induces a 1,000% increase in the early phase of GLP-1 secretion, which does not return to baseline even after 120 min (Herrmann et al., 1995). In the same study, oral ingestion of a mixed meal containing soybean oil, casein, and glucose induces a rapid ∼6-fold increase in GLP-1 levels, which is lower than corn oil alone and also leads to a return to baseline (Herrmann et al., 1995). Ileal luminal perfusion of a mixed meal in rats induces a rapid rise (2-fold) in portal plasma GLP-1 in 30 min (Herrmann et al., 1995). A 20% infusion of Intralipid in the perfused rat ileum, however, does not significantly increase portal plasma GLP-1 from baseline (Herrmann et al., 1995), suggesting that since orally ingested fatty acids do not reach the ileum, a direct sensing mechanism for this lipid composition does not exist in the ileum or that GLP-1 in this experiment bypasses portal circulation. By contrast, experiments directly administering corn oil into either duodenal or ileal luminal compartments in anesthetized rats demonstrate significantly increased plasma GLP-1 (obtained from carotid artery) to the same extent from baseline (Roberge and Brubaker, 1993). Taken together, these studies demonstrate that either higher fatty acid concentration, mechanical stimulation, or a specific blood sampling pool is required to detect this response from the distal gut. While not often measured, the GLP-1/GLP-2 ratio (detecting C-terminal of GLP-1 and N-terminal of GLP-2) remains consistent throughout an oral fat tolerance test, but interestingly significantly increases at 120 and 250 min during an OGTT in obese men (Matikainen et al., 2016). Additionally, in response to a meal, in patients with short bowel syndrome with a preserved colon (jejuno-colonic anastomosis), both baseline GLP-1 and GLP-2 are elevated with GLP-2 levels threefold greater than control patients (average concentration of 72 pmol/L), which persists throughout the post-prandial period (Jeppesen et al., 2000).

Lymph fistula experiments in rats reveal post-prandial levels in intestinal lymph are 5–6 times higher for GLP-1 compared to portal venous plasma (D’Alessio et al., 2007; Lu et al., 2007). Similarly, GLP-2 concentrations in the lymph are significantly higher (∼2-fold) than in blood at fasting and 2 h after (∼3-fold) duodenal infusion of perilla oil (Sato et al., 2013). The physiological advantage for lymph vs. blood secretion is not clear; however, DPP4 activity is significantly higher during fasting (20-fold) and post-meal (3-fold) in plasma than in lymph (D’Alessio et al., 2007). Intraduodenal infusion of Liposyn significantly increases lymph flow, lymph GLP-1 levels and secretion rates before increases in lymph TG and lymph free fatty acid (FFA) compared to saline control are observed (Lu et al., 2012). Pluronic L-81 impairs lymphatic transport of TG without inhibiting fatty acid absorption or TG assembly (Tso et al., 1981; Hayashi et al., 1990), therefore leading to the accumulation of large apical lipid droplets in enterocytes (Tso et al., 1981). The addition of pluronic L-81 to the Liposyn infusion significantly reduces lymph flow to rates observed in saline control. It completely abolishes TG and FFA concentrations and delays the peak in lymph GLP-1 concentrations from 30 to 120 min, with a 75% reduction in the rate at 30 min, but secretion was the same at 60 min (Lu et al., 2012). Overall, the addition of L-81 to Liposyn did not reduce the cumulative GLP-1 output to the same levels as saline controls, whereas GIP secretion was abolished (Lu et al., 2012).

In the presence of glucose, chylomicrons (10 and 100 μg/mL) significantly increase GLP-1 secretion from GLUTag cells, murine duodenal cultures, and human duodenal cultures (Psichas et al., 2017). Lipoprotein lipase (*Lpl*) is highly expressed in duodenal L cells and GLUTag cells; both the lipase inhibitor orlistat and siRNA-mediated knockdown of *Lpl* significantly inhibits chylomicron-induced GLP-1 secretion in GLUTag cells (Psichas et al., 2017). LPL-mediated hydrolysis of chylomicrons yields long chain fatty acids and monoacylglycerols, which are ligands for FFA1 and GPR119. Indeed, L cells express free acid receptors *Ffar1* and G-protein coupled receptor 119 (Psichas et al., 2017). FFA1 receptor signaling increases GLP-1 secretion with or without chylomicron treatment, as shown with FFA1 agonist (AM-1638), FFA1 antagonist (GW110), and siRNA-mediated knockdown experiments in GLUTag cells (Psichas et al., 2017). While GPR119 activation stimulates GLP-1 secretion in primary duodenal cultures, activation is not absolutely required for GLP-1 secretion as shown by L cell specific knockout (Psichas et al., 2017). Additionally, inhibiting both FFA1 and GPR119 at the same time does not impact GLP-1 secretion upon chylomicron treatment in primary duodenal cultures (Psichas et al., 2017). Also, orlistat does not significantly impact chylomicron-stimulated GLP-1 secretion in duodenal cultures, suggesting that LPL-mediated release of FFA1 and GPR119 ligands may be restricted to GLUTag cells (Psichas et al., 2017). However, in primary cultures, only the apical membrane of L cells are exposed to chylomicrons (Psichas et al., 2017). Therefore, basolateral LPL access to chylomicrons may be required.

In healthy men, oral glucose significantly increases plasma total GLP-1 [GLP-1(1–36) and GLP-1(7–36)] levels after 30 min; its rise is delayed compared to the rapid increase of circulating GIP (Herrmann et al., 1995). Compared to oral glucose, oral galactose and amino acids rapidly increase plasma GLP-1 levels (Herrmann et al., 1995). In healthy men, intraduodenal infusion of glucose induces a rapid 200% increase in GLP-1 that returns to baseline by 30 min (Herrmann et al., 1995). Ileal luminal perfusion of a 5% glucose dissolved in saline in rats induces a rapid rise (∼2-fold) in portal plasma GLP-1 in 30 min (Herrmann et al., 1995). This effect is lost when glucose is dissolved in distilled water (Herrmann et al., 1995). While significantly lower than portal GLP-1 secretion upon intraduodenally delivered glucose, delivering glucose directly to the stomach in anesthetized rats with a pylorus ligature induces a significant increase in portal GLP-1 [+133 pM vs. phosphate-buffered saline (PBS)] and gastric vein (+140 pM vs. PBS) at 15 min compared to PBS control, where ∼1/2 of this total GLP-1 in the gastric vein is the active peptide (Ribeiro-Parenti et al., 2021). Gastric mucosal cells produce proglucagon, GLP-1, and GLP-2 (Ribeiro-Parenti et al., 2021). Despite GLP-1 concentration being higher in the fundus than the antrum, its release *ex vivo* upon IBMX stimulation increases to the same extent in both the fundus and antrum, suggesting a significant contribution to both portal and gastric GLP-1 (Ribeiro-Parenti et al., 2021). Intraduodenal administration of sucrose, sucralose, and the artificial sweetener PALSWEET each significantly increase lymph GLP-2 output compared to saline control (Sato et al., 2013).

A paracrine relationship exists between GLP-1-secreting L cells and somatostatin-secreting D-cells (Jepsen et al., 2019). Additionally, the somatostatin receptor *Sstr5* expression is present in GLP-1-immunoreactive cells (Jepsen et al., 2019). GLP-1 secretion in response to intraduodenal infusion of glucose increases with somatostatin receptor antagonism (SSTr2 and SSTr5) (Jepsen et al., 2019). Similarly, somatostatin secretion is dependent on GLP-1R activation as its secretion is inhibited upon GLP-1R antagonist (exendin-9) treatment (Jepsen et al., 2019). Taken together, this relationship is regulated by L cell and D cell expression of SSTr5 and GLP-1R, respectively. Additionally, these findings explain the increased endogenous GLP-1 release upon exendin-(9–39) treatment.

#### Hormonal Stimulation of GLP-1 Secretion

Plasma GLP-1 levels peak within 5–15 min of food ingestion, where certainly these nutrients do not reach the ileum to directly stimulate L cells (Borgstrom et al., 1957). A neuroendocrine loop exists in proximal-distal intestine to stimulate ileal L cells when dietary fat enters the duodenum (Roberge and Brubaker, 1993; Rocca and Brubaker, 1999). As previously mentioned, administration of corn oil to duodenal luminal compartments elicits the same plasma GLP-1 response compared to corn oil administration to ileal luminal compartments (Roberge and Brubaker, 1993). Despite the presence of L cells in the duodenum, they are not responsible for the GLP-1 release as removing the jejunum-ileum before infusing the duodenal compartment with fat prevents the observed increase of plasma GLP-1 (Roberge et al., 1996). Still, plasma GIP secretion in response to duodenal luminal administration occurs earlier than GLP-1 secretion (Roberge and Brubaker, 1993). Importantly, intravenous infusion of post-prandial levels of GIP increases plasma GLP-1 levels twofold, independent of blood glucose levels (Roberge and Brubaker, 1993), suggesting that GIP stimulates early GLP-1 secretion in response to duodenal luminal nutrients. Indeed, GLP-1 secretion is abolished upon corn oil infusion to the proximal duodenal compartment in vagotomized rats (Rocca and Brubaker, 1999). Electrical stimulation of the vagus nerve stimulates GLP-1 secretion, even in the absence of nutrients (Rocca and Brubaker, 1999). GIP can stimulate the first phase of GLP-1 secretion independent of the vagus nerve, but only when infused at suprapharmacological levels, as evidenced by the rapid rise and fall in plasma GLP-1 upon supraphysiological infusion of GIP in sham and vagotomized rats (Rocca and Brubaker, 1999). At physiological levels, infusion of GIP does not stimulate GLP-1 secretion in vagotomized rats compared to the peak observed at 10 min in the sham controls (Rocca and Brubaker, 1999). Curiously, ingestion of 200 mL of pure water increases late phase plasma GLP-1, while GIP secretion is unchanged (Herrmann et al., 1995), suggesting a GIP-independent and potentially mechanically-mediated increase in GLP-1.

Leptin increases GLP-1 secretion in fetal rat intestinal cells, GLUTag, and NCI-H716 human enteroendocrine cells, all of which express a functional leptin receptor in GLP-1+ cells (Anini and Brubaker, 2003). Leptin (1 mg/kg, *i.p.*) increases fasting GLP-1 secretion 1.8-fold compared to saline control, reaching 6 pmol/L at 120 min, which increases even further in leptin-deficient mice (*ob/ob*) (Anini and Brubaker, 2003). Therefore, leptin appears to induce the later phase of GLP-1 secretion compared to the early peak upon GIP treatment, which may be important for potentiating the leptin-stimulated reduction in food intake. Interestingly, leptin treatment significantly increases water intake in healthy rats (Sivitz et al., 1997), which may link the late-phase GLP-1 secretion induced by both leptin and water. Additionally, while high-fat fed mice with leptin resistance display increased GLP-1 content in the ileum and the colon, both fasting and glucose-stimulated GLP-1 secretion are significantly reduced in these mice (Anini and Brubaker, 2003), which may provide a link between leptin resistance in L cells and the reduced late phase (60–160 min) total and active GLP-1 secretion in patients with diabetes compared to healthy individuals (Vilsboll et al., 2001).

Similar to K cells, L cells also express the Galinin receptor, GAL_1_, and its activation via Galinin treatment or GAL_1_ agonist (M617) treatment prevents the accumulation of cyclic adenosine monophosphate (cAMP) in L cells within primary duodenal cultures in response to the adenylyl cyclase activator, forskolin and inhibits GLP-1 secretion from primary duodenal and ileal cultures (Psichas et al., 2016).

#### Inflammation and GLP-1 Secretion

Links between inflammation, the gut microbiota and GLP-1 secretion have also been reported (Everard et al., 2011; Greiner and Backhed, 2016; Wu et al., 2018; Covasa et al., 2019; Martchenko et al., 2020). Indeed, lipopolysaccharide (LPS) acutely induces GLP-1 secretion (Nguyen et al., 2014). This was demonstrated to be dose- and time-dependent, where LPS-induced increases in circulating IL-6 (30 min) preceded that of both total and active GLP-1 (120 min) (Kahles et al., 2014). LPS also stimulates the release of IL-1β, where the latter also increases plasma GLP-1 upon *i.p.* injection in mice to a greater extent than IL-6 injection (Kahles et al., 2014). However, loss of IL-1R signaling does not impact LPS-mediated GLP-1 secretion, as shown in *Il1r^–/–^* mice while neither LPS nor IL-1β stimulate GLP-1 secretion in *Il6^–/–^* mice (Kahles et al., 2014). Similarly, IL-6, but not LPS or IL-1β, increases GLP-1 secretion from GLUTag cells (Kahles et al., 2014). LPS induces GLP-1 secretion to the same extent in both the fasted and fed state, where not surprisingly, insulin is only increased in these mice during the fed state. While these data demonstrate the glucose-dependency for the insulinotropic role of GLP-1, they also reveal nutrient-independent GLP-1R signaling pathways (Kahles et al., 2014). Plasma total GLP-1 concentrations are significantly higher in patients with sepsis than non-septic ICU patients; these levels are positively associated with IL-6, C-reactive protein, and the association of GLP-1 with plasma insulin is lost (Kahles et al., 2014). Taken together, this study reveals an integral role for the gut in systemic inflammation in pathways that remain incompletely understood.

Hwang et al. (2015) demonstrate that the antibiotics, vancomycin and bacitracin decrease the abundance of both Bacteroidetes and Firmicutes, and increase Proteobacteria, which is associated with increased GLP-1 secretion and improved glucose tolerance and insulin resistance. *Coriobacteriaceae* are involved in the metabolism of bile acids. This family of bacteria are able to metabolize primary bile acids into secondary bile acids, which then bind to TGR5 and stimulate GLP-1 secretion (Allin et al., 2015). Fourteen weeks of HFD-feeding supplemented with *Akkermansia muciniphila* significantly increases the ileal expression of *Gcg* and *Pcsk1*, and oral glucose-stimulated plasma GLP-1 compared to mice fed the HFD alone (Yoon et al., 2021). The cell-free supernatant from live *A. muciniphila* isolated from human feces significantly increases GLP-1 secretion from human enteroendocrine L cells (NCI-H716) in a dose-dependent manner and to a greater extent than the microbial products acetate and propionate (Yoon et al., 2021). Indeed, the authors identified, the protein P9 of the peptidase S41A family robustly increases GLP-1 secretion from human L cells *in vitro* and in mice after a single *i.p*. injection compared to saline control and injection of SCFA (Yoon et al., 2021). Mice fed a HFD supplemented with P9 display increased ileal *Gcg* and *Pcsk1* expression as well as compared to mice fed the HFD-alone (Yoon et al., 2021). HFD-fed mice supplemented with *A. muciniphila* also display increased ileal and colonic *Il-6* mRNA expression, and while IL-6 treatment in GLUTag cells does not stimulate GLP-1 secretion to the same extent as P9, co-treatment of IL-6 and P9 induces an additive effect (Yoon et al., 2021). Interestingly, P9 supplementation to a HFD does not increase plasma GLP-1 in *Il6^–/–^* mice (Yoon et al., 2021). A far lesser amount of studies have correlated populations of microbiota with GLP-2 secretion (Utzschneider et al., 2016). Already known to increase GLP-1 secretion, ingestion of *Lactobacillus reuteri* demonstrates increased GLP-2 secretion as well (Simon et al., 2015).

#### Exercise-Induced GLP-1 Secretion

Ninety minutes of exercise in mice induces a 2.5-fold increase in plasma active GLP-1, mediated by skeletal-muscle-derived IL-6, as shown by abolishing exercise-induced active GLP-1 levels in *Il6^–/–^* mice and by treating wild-type mice with an antibody to IL-6 (Ellingsgaard et al., 2011). Interestingly, injecting mice with 400 ng of recombinant mouse IL-6 twice daily for 7 days significantly increases fasting plasma active GLP-1, as well as ileal *Gcg* and *Pcsk1* mRNA, but not plasma GLP-2 (ELISA) or DPP4 activity (Ellingsgaard et al., 2011). Indeed, GLUTag cells express the IL-6 receptor, and IL-6 treatment increases GLP-1 secretion in a dose-dependent manner, where acute IL-6 treatment increases GLP-1 exocytosis in a JAK2-STAT3-dependent manner, and chronic IL-6 treatment increases GLP-1 content and glucose uptake in a sodium glucose transporter 1-dependent manner in the L cell (Ellingsgaard et al., 2011). Surprisingly, despite increasing *Gcg* mRNA, chronic IL-6 treatment does not increase plasma GLP-2 levels suggesting a difference in GLP-1 and GLP-2 transcript or protein stability.

## Receptor Expression Within the Gastrointestinal Tract

GPCRs initiate the cellular responses to nearly all hormones and neurotransmitters; they are grouped into six main classes (A to F) by sequence homology and function. GCPRs have 7 transmembrane helices, and in the cases of GIPR, GLP-1R and GLP-2R, signal via Gs-mediated cAMP production and downstream signaling cascades. They are all class B1 GPCRs, share significant sequence similarity (Usdin et al., 1993) and form secretin-VIP receptor family (Campbell and Scanes, 1992).

### GIPR Expression

The human GIP receptor (GIPR) gene is ∼13.8 kb long containing 14 exons. The receptor is 466 amino acids in length, including a signal peptide and 7 transmembrane domains; the gene contains 14 exons (Yamada et al., 1995). The first 92 bp of the *GIPR* gene contains 88% sequence identity between rat and human; interestingly, neither promoter regions contains a TATA box (Boylan et al., 2006). MZF1/Sp1-C (−75), Sp1-B (−57), and Sp1-A (−45) transcription factor binding sites were identified using radiolabeled synthetic probes and confirmed with CHiP analysis (Boylan et al., 2006). Indeed, sequence deletion between −85 and −40 decreases promoter activity by 88% (Boylan et al., 2006).

The identification of cell-specific expression of *Gipr* in the gastrointestinal tract remains largely unsolved; however, clues are beginning to emerge. On a whole tissue level, *Gipr* mRNA is expressed in rat stomach, duodenum, and proximal small intestine (Usdin et al., 1993; Coon et al., 2013). *GIPR* mRNA expression is detected in neuroendocrine tumors isolated from the small bowel and colorectal tumors (Sherman et al., 2013; Koehler et al., 2015) (Table 1). GIPR is faintly detected at the protein level at multiple sizes (50, 55, 60, and 70 kDa) in jejunal mucosal cells compared to the strong signal at 50 kDa in pancreatic homogenates (Coon et al., 2013). In this same study, GIPR immunohistochemistry demonstrated positive staining beneath the basolateral surface of epithelial cells of the proximal jejunum (Coon et al., 2013). In the stomach, RNAseq of purified gastric somatostatin-producing D-cells from SST-Cre.ROSA26^EYFP^ mice reveal *Gipr* expression in these cells (Adriaenssens et al., 2015). A number of distinct neuronal populations also express the *Gipr* (Adriaenssens et al., 2019). Genetic elimination of *Gipr* in hematopoietic cell lineages, including endothelial cells (*Gipr^*Tie2*–/–^* mice) does not impact jejunal *Gipr* mRNA (Pujadas et al., 2020).

**TABLE 1:** Summary of methods used to identify GIPR expressing cells within the gastrointestinal tract.

Cell/organ	Species	Method of identification	References
Small bowel neuroendocrine tumors	Human	*GIPR* mRNA qRT-PCR analysis	Sherman et al., 2013
Human colorectal tumors	Human	*GIPR* mRNA qRT-PCR analysis	Koehler et al., 2015
Human hypothalamic cells (vascular, glial, neuronal cells)	Human	Single-cell RNA sequencing of GIPR+ cells	Adriaenssens et al., 2019
T-cells, myeloid cells, myeloid precursors	Mouse	*Gipr* mRNA RT-qRT-PCR analysis	Pujadas et al., 2020

### GLP-1R Expression in the Gut

The transcriptional start site of the GLP-1R does not contain a TATA- or a CAAT-box element, however, it contains 3 putative Sp1 binding sites (Lankat-Buttgereit and Goke, 1997). Within the 350 bp region, 74% of the sequence is GC nucleotides (Lankat-Buttgereit and Goke, 1997). *Glp1r* expression determined by RNAscope *in situ* hybridization reveals the highest expression in duodenal Brunner’s glands and in stomach gland parietal cells (Wismann et al., 2017). Consistent with this use of a reporter mouse together with a number of validation approaches the GLP-1R was identified in chief cells, parietal cells and Brunner’s glands (Andersen et al., 2021). A well-validated antibody to the GLP-1R (MAb 3F52) and corroborated with ^125^I-labeled GLP-1 also demonstrated a strong signal in stomach parietal cells, basolateral epithelial cells in the duodenum, Brunner’s glands and the myenteric nerve plexus (Pyke et al., 2014). *Glp1r* expression localizes to the basolateral side of enterocytes in the mucosal layer, and its abundance increases distally (Wismann et al., 2017). *Glp1r* mRNA is higher in mucosal cells from the ileum and colon than in the non-epithelial fraction (Kedees et al., 2013). Conversely, *Glp1r* mRNA expression is highest in the jejunum within the epithelial fraction, followed by ileum then colon (Kedees et al., 2013). *Glp1r* is not detected in GLP-1+ cells (L cells) (Grigoryan et al., 2012); however, it is detected in chromogranin A+ enteroendocrine cells (Kedees et al., 2013; Andersen et al., 2021). *Glp1r* is also detected in Paneth cells, identified by lysozyme expression, in the jejunum and ileum crypts but not colon, distinct from proliferating Ki67+ cells (Kedees et al., 2013). *Glp1r* mRNA expression increases with age from 2 to 12 weeks in murine jejunum, ileum, and colon (Campos et al., 1994). Additionally, *Glp1r* expression in mice is detected in a subset of neurons of the myenteric and submucosal plexus (Andersen et al., 2021) that also express the neuron cytoplasmic protein 9.5 (PGP9.5) (Kedees et al., 2013) (Table 2 and Figure 1).

**TABLE 2:** Summary of methods used to identify GLP-1R expressing cells within the gastrointestinal tract.

Cell/organ	Species	Method of identification	References
Human colorectal tumors	Human	*GLP-1R* expression (qRT-PCR analysis of RNA).	Koehler et al., 2015
Intestinal intraepithelial lymphocyte (IEL)	Mouse	*Glp-1r* Real-time qRT-PCR (mRNA), immunohistochemistry (rabbit polyclonal anti-CD3 antibody and hematoxylin). Used GLP-1R^–/–^ model. *Glp-1r* transcript identified in isolated RNA (qRT-PCR), southern blot detects *Glp-1r* PCR product.	Yusta et al., 2015
Synaptic type neurons	Mouse	GLP-1R fluorescent cell population. Whole cell current clamp.	Richards et al., 2014
After-hyperpolarizing type neurons	Mouse	GLP-1R fluorescent cell population. Whole cell current clamp.	Richards et al., 2014
Inhibitory motor neurons	Mouse	Immunostained for nNOS (marker mainly restricted to inhibitory motor neurons), most GLP-1R fluorescent neurons were nNOS+.	Richards et al., 2014
Intrinsic primary afferent neurons	Mouse	GLP-1R-fluorescent cells in culture stained for Calretinin (marker for intrinsic primary afferent neurons)	Richards et al., 2014
Vagal afferent neurons	Mouse	GLP-1R-fluorescent cells. Immunostained for RFP.	Richards et al., 2014
Intraepithelial lymphocytes	Mouse	*Glp1r.tdTomato* reporter mouse. ISH of GLP-1R and tdTomato expression. *Glp-1r* mRNA *in situ* hybridization.	Andersen et al., 2021
Neurotensin+ N-cells, Somatostatin+ D-cells, PYY+ L-cells, serotonin+ enterochromaffin cells (EC)	Mouse	*Glp1r.tdTomato* reporter mouse. ISH of GLP-1R and tdTomato expression. *Glp-1r* mRNA *in situ* hybridization.	Andersen et al., 2021
Mucus cells (antrum)	Mouse	*Glp1r.tdTomato* reporter mouse. ISH of GLP-1R and tdTomato expression. *Glp-1r* mRNA *in situ* hybridization.	Andersen et al., 2021
Parietal cells	Mouse	Td.Tomato-positive cells. ISH of GLP-1R and tdTomato expression. *Glp-1r* mRNA *in situ* hybridization.	Andersen et al., 2021
Chief cells	Mouse	Td.Tomato-positive cells. Immunohistochemistry.	Andersen et al., 2021
αβ, γδ T cells	Mouse	Expression of *Glp-1r* (mRNA).	He et al., 2019
Myenteric neurons	Mouse	*Glp1r*-CRE fluorescent reporter.	Richards et al., 2014
Neurons of the myenteric and submucosal plexus	Mouse	Expression of *Glp-1r* (mRNA). Immunohistochemistry.	Kedees et al., 2013
Brunner’s gland (duo)	Mouse	Glp-1r.tdTomato signal. ISH of GLP-1R and tdTomato expression. *Glp-1r* mRNA *in situ* hybridization.	Andersen et al., 2021
Parietal cells Brunner’s gland	Monkey	MAb 3F52.	Pyke et al., 2014
Parietal cells Brunner’s glands	Mouse	RNAScope *in situ* hybridization.	Wismann et al., 2017
Myenteric nurons	Monkey	MAb 3F52	Pyke et al., 2014
Epithelial cells	Mouse	Expression of *Glp-1r* (mRNA). Immunohistochemistry.	Kedees et al., 2013
Basolateral epithelial cells	Monkey	MAb 3F52.	Pyke et al., 2014

Studies using the mouse *Glp1r* promoter to drive expression of a fluorescent reporter protein reveal *Glp1r* expression in the antral area of the stomach (near the gastric pylorus) as a fibrous signal that does not overlap with smooth muscle α-actin (αSMA) (Richards et al., 2014). *Glp1r* expression is also observed in the arteries and arterioles of the intestine and colocalizes with αSMA and the pericyte marker NG2 (Richards et al., 2014) (Figure 1). In this model, *Glp1r* fluorescence is absent from the epithelial layer. Instead, mRNA expression is detected in myenteric ganglia in the intestinal mucosa, which are excitable by GLP-1 treatment *ex vivo* (64% synaptic and 36% after-hyperpolarizing types) (Figure 1 and Table 2). Indeed, 63% of *Glp1r*-fluorescent neurons in primary small intestinal cultures and 19% in colonic cultures are neuronal nitric oxide synthase (nNOS) positive markers for inhibitory motor neurons (Richards et al., 2014). GLP-1 receptors are expressed in the enteric nervous system and in the vagus nerve (Grasset et al., 2017), which allow for the activation of the gut-brain-periphery axis. As such, the presence of GLP-1R+ cell bodies in the enteric nervous system has been proposed to provide the signaling route to the central nervous system (CNS) required for distally secreted GLP-1. Consistent with *Glp1r* mRNA expression, immunofluorescence analyses in Glp1r.tdTomato reporter mice reveal GLP-1R expression in various enteroendocrine cells (Table 1), but not GLP-1+ cells (Andersen et al., 2021). Sequential collagenase digestion of the gut reveals *Glp1r* expression to be within the epithelial fraction instead of crypt, mesenchyme, or smooth muscle layer fractions (Yusta et al., 2015). Within the epithelial compartment of the small intestine, intraepithelial lymphocytes (IELs) (both the Tαβ and Tγδ subsets) express *Glp1r* (Yusta et al., 2015; He et al., 2019) (Figure 1 and Table 2). Additionally, GLP-1R-expressing αβ and γδ T cells transit to the gut via integrin B7 (*Itgb7*) (He et al., 2019). Indeed, IELs encode a functional GLP-1R as exendin-4 treatment in sorted activated and non-activated IELs increases cAMP levels. However, GLP-1R in IELs is not required for IEL development or recruitment to the gut as their abundance does not change in response to GLP-1R agonist treatment or in *Glp1r^–/–^* mice (Yusta et al., 2015). These receptors are functional as mice receiving exendin-4 *i.v.* exhibit an 84% increase in *c-fos* mRNA expression in the ileal mucosa (Kedees et al., 2013). The increased c-fos expression occurs in neurons as it is abolished upon co-treatment with tetrodotoxin, a voltage-gated sodium current blocker. Additionally, exendin-4 treatment increases *c-fos* expression in in GLP-1R+ Paneth cells (Figure 1), which is abolished when exendin-(9–39) is administered prior to exendin-4 treatment (Kedees et al., 2013).

### GLP-2R Expression in the Gut

The human GLP-2 receptor is localized to chromosome 17p13.3 and encodes a 550 amino acid G protein-coupled receptor, processed to become a 486 amino acid receptor (Munroe et al., 1999). The gene at chromosome 17p13.3 encoding for the human GLP-2 receptor is also very well conserved as the rat sequence is 80% of the same amino acid sequence (Shin et al., 2005). The GLP-2 receptor is 14 exons long and has seven transmembrane domains and, although similar in amino acid sequence to both the glucagon and GLP-1 receptor, only recognizes GLP-2 and not related members of the glucagon family (Drucker and Yusta, 2014).

GLP-2R is expressed in the gastric mucosa in a subpopulation of fundas gland cells (Li et al., 2017) (Table 3). In the rat jejunum, *Glp2r* expression as a percentage of the expression in intact intestine is 0.07, 33, 256, and 392% in the epithelium, mucosa, smooth muscle layer, and the intestine devoid of epithelium, respectively (Pedersen et al., 2015). GLP-2R transcripts are expressed in human colorectal tumors (Koehler et al., 2015) and GLP-2R protein is expressed in human colon neoplasms (Koehler et al., 2008) (Table 3). In rats, mice, marmosets and human intestinal tissue, GLP-2R localizes to cells residing immediately below the basolateral membrane of enterocytes, which are subepithelial myofibroblasts as marked by αSMA (Orskov et al., 2005) (Figure 1 and Table 3). *Glp2r* expression is most abundant in the lamina propria of duodenal and jejunal villi (Wismann et al., 2017), where in the jejunum its expression within the lamina propria stromal cells predominate in the upper half of villi (Yusta et al., 2019) (Figure 1). The receptor’s location in the lamina propria is consistent with evidence that suggests the link between GLP-2 to KGF, IGF-1, and ErbB, as these growth factors are produced and secreted from stromal cells found in the lamina propria (Yusta et al., 2019). GLP-2R protein in neonatal pigs colocalizes with chromogranin A+ enteroendocrine cells in the jejunal villus (∼58%) and crypt epithelium (60%) (Guan et al., 2006) (Table 3). A rat polyclonal antibody localized using immunohistochemistry the GLP-2R to vagal afferents, enteric neurons, enteroendocrine cells, and myenteric plexus nerve fibrils (Nelson et al., 2007). Isolated rat intestinal mucosal cells expressing *Glp2r* transcripts also expressed markers for enteroendocrine or neural cells (Walsh et al., 2003) (Table 3). Isolated Human GLP-2R protein also colocalizes to chromogranin A+ enteroendocrine cells in both the villus and crypt epithelium (Guan et al., 2006). Human GLP-2R protein colocalizes to 5-HT-containing cells in the epithelium, a neurotransmitter released by enteroendocrine cells (Guan et al., 2006). Human VIP+ enteric neurons in the submucosal plexus and myenteric plexus express the GLP-2R (Guan et al., 2006) (Table 3). In the mouse duodenal myenteric plexus, ∼18% of GLP-2R+ are nNOS+, 10% are vasoactive intestinal polypeptide (VIP)+, ∼71% are choline-acetyltransferase (ChAT)+, and 27% are SP+ (Cinci et al., 2011) (Figure 1 and Table 3). In the submucosal plexus, only SP+ cells were GLP-2R+ (Cinci et al., 2011) (Figure 1 and Table 3). Human eNOS+ enteric neurons in the submucosa also express the GLP-2R, supporting a direct role for GLP-2-mediated increase in eNOS protein and NOS release through cAMP-dependent protein kinase A (Guan et al., 2006) (Table 3).

**TABLE 3:** Summary of methods used to identify GLP-2R expression throughout the gastrointestinal tract.

Cell/organ	Species	Method of identification	References
Human colorectal tumors	Human	*GLP-2R* mRNA transcripts expressed qRT-PCR.	Koehler et al., 2015
Human colon neoplasms	Human	Immunohistochemistry.	Koehler et al., 2008
Gastric chief cells	Human	GLP-2R Fluorescence ISH. GLP-2R by western blot.	Li et al., 2017
Myenteric plexus	Human	*In vitro* receptor autoradiography of human intestinal tissue.	Pedersen et al., 2015
Lamina propria stromal cells	Mouse	ISH with RNAscope, *Glp-2r* mRNA detected. GLP2R-driven LacZ expression.	Yusta et al., 2019
Vagal afferents	Rat	GLP-2R antibody localizing GLP-2R immunoreactivity. ISH.	Nelson et al., 2007
Intestinal muscularis	Mouse	*Glp-2r* mRNA transcripts by RT-PCR.	Shin et al., 2005
Jejunal enteroendocrine cells	Pig	*Glp-2r* mRNA transcripts by qRT-PCR of laser micro-dissected tissue. *In situ* hybridization. Immunostaining.	Guan et al., 2006
Subepithelial myofibroblasts	Mouse Rat Mouse	*Glp-2r* mRNA expression by qRT-PCR. Immunohistochemistry (antibody 99077).	Orskov et al., 2005
Subepithelial myofibroblasts	Mouse Rat Mouse	*Glp-2r* mRNA expression by qRT-PCR. Immunohistochemistry (antibody 99077).	Orskov et al., 2005
Isolated intestinal mucosal cells	Rat	GLP-2R mRNA transcripts by RT-PCR.	Walsh et al., 2003
Lamina propria of duodenal and jejunal villi, submucosal nerve plexuses	Mouse	RNAScope *in situ* hybridization.	Wismann et al., 2017
Smooth muscle layer, intestine devoid of epithelium, respectively	Rat	*Glp-2r* mRNA expression by qRT-PCR.	Pedersen et al., 2015

## Distinct and Overlapping Functional Roles of GIPR, GLP-1R, and GLP-2R

### Regulation of Post-prandial Lipid Metabolism

Spearman correlations between GLP-1, GLP-2, GIP, and TG responses in plasma during an oral fat tolerance test in obese men reveal a small albeit significant positive correlation (*r*-squared values close to zero) between all three hormone area under curves (AUCs) for TG and apoB48 (Matikainen et al., 2016). In this study, these gut hormones display small contributions to explaining the variance in TG AUC, where instead fasting TG values serve as the largest contributor for explaining this variance (Matikainen et al., 2016). Still, the high concentrations of GLP-1, GLP-2, and GIP within the gut circulation relative to systemic circulation suggest that endogenous gut hormone action on chylomicron secretion may be local and underestimated.

#### GIP Receptor (GIPR)

Chronic reduction in GIP secretion reduces obesity and insulin resistance in high-fat fed mice (Nasteska et al., 2014). Interestingly, dietary fat absorption and intestinal-TG secretion are unchanged upon K cell destruction (Pedersen et al., 2013; Holst et al., 2016). Similarly, GIP infusion *i.v.* does not impact TG levels (Holst et al., 2016). Rather, GIP has been shown to increase circulating lipid clearance via an increase in adipose tissue blood flow. GIPR antagonist, GIP(3–30)NH2, and GIP co-infusion in lean individuals prevented a fivefold increase in adipose tissue blood flow induced by GIP infusion alone (Asmar et al., 2017). Additionally, both TG and glucose uptake decrease in response to GIP(3–30)NH2 alone and GIP co-infusion compared to GIP infusion alone (Asmar et al., 2017).

Co-administration of triton-WR1339 infusion and D-Ala2-GIP injection 20 min following oil gavage in mice significantly increases TG accumulation in plasma at 60 and 90 min, and ApoB-48 levels at 90 min compared to PBS control (Hsieh et al., 2010), suggesting a role for GIP in plasma TG independent of triglyceride rich lipoprotein (TRL) clearance. Additionally, selective deletion of *Gipr* in brown adipose tissue significantly increases both fasting (overnight) and fed (1h re-feed) TG levels of high-fat fed mice (Beaudry et al., 2019). Furthermore, acute lipid challenges in *Gipr^*BAT*–/–^* mice fed a high-fat diet for 8–10 weeks housed at room temperature reveal significantly increased TG excursion, an effect lost upon 28 weeks of high-fat feeding (Beaudry et al., 2019). GIP, in the presence of insulin, increases LPL gene expression in 3T3-L1 adipocytes via PKB/LKB1/AMPK signaling (Kim et al., 2007a) mediated by resistin (Kim et al., 2007b) and in human adipocytes by increasing TORC2 and phospho-CREB nuclear localization to bind to the CRE-II promoter region (Kim et al., 2010). GIP infusion significantly increases LPL activity in obese (*fa/fa*) and lean (*fa/-*) VDF Zucker rats (Kim et al., 2007a). Conversely, treatment of rats with the GIPR antagonist, rat GIP (3–30)NH2, does not modify food intake but significantly increases plasma TG and LPL compared with controls (Baldassano et al., 2019). Alternatively, D-Ala2-GIP treatment significantly reduces serum LPL activity in both chow- and high-fat diet-fed mice (Szalowska et al., 2011). However, the significance of endogenous GIP secretion as a dominant regulator of LPL secretion is uncertain. In humans, intravenous infusion of a somatostatin analog, octreotide, 30 min prior to carbohydrate meal (Hycal) significantly impairs insulin, GLP-1, and GIP secretion in both lean and obese women, yet post-heparin LPL activity (contributions from adipose, skeletal and cardiac tissue) is unchanged 1.5 h post-peak insulin in lean and obese women (Ranganath et al., 1999). Therefore, suppression of insulin, GIP, and GLP-1 does not impact plasma LPL activity following oral carbohydrate.

#### GLP-1R

High-fructose feeding for 10 days in hamsters significantly increases plasma TG and cholesterol levels (Hsieh et al., 2010), where only the former can be significantly decreased after 3 weeks of systemic DPP4 inhibition (sitagliptin). This treatment paradigm reduces post-prandial TRL-fraction TG levels and ApoB48 production (Hsieh et al., 2010). Acute sitagliptin administration to chow-fed mice significantly reduces plasma cholesterol and TG at 90 min post-triton infusion and oil gavage (Hsieh et al., 2010). Co-administration of triton by infusion and exendin-4 by injection 20 min following oil gavage in mice significantly decreases TRL-fraction TG accumulation at 90 min, and ApoB48 levels at 60 and 90 min, an effect significantly reversed by the co-administration of GLP-1R antagonist exendin(9–39) 20 min prior to gavage (Hsieh et al., 2010). While sitagliptin and exendin-4 significantly increase plasma insulin levels 5-min post injection, these levels are not significantly different from PBS control after 20 min, suggesting that the GLP-1 mediated reduction in intestinal-TG secretion is independent of the incretin effect. Indeed, the authors show that in co-administration of insulin injection and triton infusion 20 min post-olive oil gavage in mice does not significantly change the accumulation of TG in plasma (Hsieh et al., 2010). This effect is significant given the studies in humans where acute insulin treatment inhibits intestinal lipoprotein secretion in response to hourly meals, an effect partially lost upon concomitant Intralipid and heparin infusion (Pavlic et al., 2010), suggesting mediation by FFA. Exendin-4 decreases TG and cholesterol in the VLDL/chylomicron fraction of chow-fed hamsters while a GLP-1R antagonist increases ApoB48 accumulation 120 min-post oil in chow-fed hamsters (Hsieh et al., 2010). Despite similar gastric emptying rates between *Glp1r^–/–^* mice wild-type controls (Baggio et al., 2004), *Glp1r^–/–^* mice display significantly increased TG accumulation in plasma and the TRL fraction as well as TRL ApoB48 post-oil gavage (Hsieh et al., 2010). Furthermore, pulse-chase experiments in primary suspended villi from chow-fed hamsters reveal that exendin-4 does not change cellular ApoB48 levels, but significantly decreases ^35^S-labeled ApoB48 secretion in the media (Hsieh et al., 2010).

Patients with type 2 diabetes treated with metformin and the GLP-1R agonist exenatide for 1 year display significantly reduced circulating TG, apoB48, and FFA following an early meal (50 g of fat, 75 g of carbohydrates, 35 g of protein). Interestingly, TG and apoB48 levels rapidly rise in the 2 h following the second meal in these patients to levels similar as pre-treatment responses (Bunck et al., 2010). In patients with recent-onset type 2 diabetes, subcutaneous injection of exenatide immediately prior to meal consumption (5,384 kJ) significantly reduces serum insulin at 2, 4, 6, and 8 h post-meal (Schwartz et al., 2010). Moreover, exenatide reduces post-meal serum TG and remnant lipoprotein TG at 2-, 4-, and 6-h post-meal, in particular preventing the 4-h peak in TG seen in placebo controls (Schwartz et al., 2010). Plasma remnant lipoprotein cholesterol is also significantly reduced 4 h post-meal in these patients (Schwartz et al., 2010). Similarly, exenatide significantly reduces serum apoB48 levels throughout the 8-h sampling period (Schwartz et al., 2010). Meal-induced increases in plasma apoCIII are also prevented by exenatide (Schwartz et al., 2010). Two weeks of exenatide treatment twice daily, 1 h before morning and evening meals, significantly reduces plasma TG following these meals (∼50% carbohydrate, 20% protein, and 30% fat) compared to placebo but TG levels rise to similar levels as placebo following the midday meal, where no changes in post-prandial FFA concentrations are observed (Schwartz et al., 2008). Exenatide treatment and co-infusion of d3-leucine 5 h after starting continuous infusion of lipid/carbohydrate formula in healthy fasted humans via nasoduodenal tube 2 h after starting a pancreatic clamp does not significantly affect plasma TG, FFA, or TRL-TG compared to placebo (Xiao et al., 2012). However, this treatment paradigm demonstrates the acute reduction in apoB48 concentrations in the TRL fraction for 10 h post-injection (−37%) compared to placebo controls with a significant decrease in apoB48 production rate, no change in fractional catabolic rate, and no changes in hepatic apoB100 levels were observed (Xiao et al., 2012). Still, the precise mechanisms through which GLP-1R signaling controls post-prandial lipid metabolism remain unclear.

Genetic elimination of *Itgb7* in mice decreases the expression of *Glp1r* on αβ and γδ T cells yet increases fasting plasma GLP-1, intestinal *Gcg* mRNA expression, and ileal L cell abundance (He et al., 2019). Interestingly, these mice display improved lipid tolerance (He et al., 2019). *In vitro* experiments reveal GLP-1 concentration in media after 24 h of co-incubation of GLUTag cells with αβ and γδ T cells negatively associate with the level of *Glp1r* expression in the latter cells (He et al., 2019). Moreover, high *Glp1r* expressing αβ and γδ T cells can further decrease GLP-1 concentration in media from GLUTag cells in the presence of exendin-4, suggesting that *Glp1r-*expressing αβ and γδ T cells act as a sink for local GLP-1 production (He et al., 2019). Additionally, this supports the increased circulating GLP-1 levels observed in *Glp1r^–/–^* mice (Lamont et al., 2012), albeit the intact receptor is required for improved post-prandial lipid tolerance. These results are replicated *ex vivo*, where media GLP-1 concentration from ileal tissue from *Itgb7^–/–^* mice is significantly higher than in the media from wild-type tissue, and this increase can be replicated in wild-type tissue upon GLP-1R antagonist (exendin-9) treatment (He et al., 2019). Overall, this additional pool of GLP-1 during fasting clearly plays an important role in GLP-1R-mediated control of circulating lipoproteins, suggesting that the circuit engaged occurs within the gut.

#### GLP-2R

Subcutaneous injection (15,000 μg) of GLP-2 5 h after the start of a liquid mixed macronutrient formula infusion through a nasoduodenal tube in healthy men significantly increases peak plasma TG and TRL-apoB48 at 1 h and area under the concentration curve for the first 3 h of treatment (Dash et al., 2014). GLP-2 does not increase TRL apoB48 by increasing the synthesis of new particles, nor does it decrease the clearance of TRL apoB48, rather, GLP-2 stimulates the release of pre-formed TRL apoB48 during the first hour of treatment (Dash et al., 2014). Similarly, GLP-2 treatment significantly increases plasma TG, TRL-TG, TRL retinyl palmitate, and retinyl palmitate in the chylomicron fraction for 2 h when administered 7 h after a meal containing retinyl palmitate (Dash et al., 2014).

*Glp2r^–/–^* mice display increased fasting and 10 min post-olive oil gavage plasma active GLP-1 compared to wild-type controls, despite similar fasting DPP4 activity levels in circulation (Fuchs et al., 2020). Accordingly, plasma-TG excursion following the olive oil gavage is not significantly different from wild-type controls, although trends for decreased secretion are observed (Fuchs et al., 2020). When administered 20 min after the oil gavage, GLP-2 increases TRL-TG and TRL-cholesterol 3.5- and 3-fold, respectively, in hamsters (Hsieh et al., 2009). Radiolabeled gavage experiments (^3^H-triolein) reveal that GLP-2 increases the radiolabel incorporation into plasma TG at 60- and 90-min post-gavage with no differences observed in plasma cholesterol compared to control (Hsieh et al., 2009). Similar to hamsters, GLP-2 treatment significantly increases plasma TG concentration at 60- and 90-min post-oil gavage as well as TG and apoB48 accumulation in the chylomicron fraction of plasma in the presence of triton WR-1339 (blocking lipoprotein catabolism) (Hsieh et al., 2009). GLP-2 does not increase the protein expression of FATP4 or MTP, rather it significantly increases the expression of glycosylated CD36. CD36 localizes to the apical membrane of enterocytes found on the tips of jejunal villi (Hsieh et al., 2009). Assessing the requirement of CD36 for GLP-2-mediated increases in intestinal-TG secretion are complicated by the increased fatty acid absorption (as shown by appearance of radiolabel in plasma), TRL-TG and TRL-apoB48 secreted by *Cd36^–/–^* mice compared to wild-type controls (Hsieh et al., 2009). Still, GLP-2 does not increase TRL-TG or TRL-apoB48 secretion in *Cd36^–/–^* mice compared to saline control (Hsieh et al., 2009). ^35^S-methionine pulse-chase experiments of jejunal fragments isolated hamsters 1 h after an olive oil gavage reveal that GLP-2 treatment *ex vivo* increases the secretion of the radiolabelled-apoB48 into the media with unchanged cellular concentrations. However, since the GLP-2 treatment *ex vivo* was for 45 min (Hsieh et al., 2009), and that GLP-2 treatment rapidly induces the mobilization of pre-formed chylomicrons by 1 h treatment in humans (Dash et al., 2014), this increase in apoB48 synthesis may be driven by clearing the preformed particles earlier than vehicle controls. Still, this experiment demonstrates that GLP-2R-expressing cell(s) mediating this indirect increase reside near enterocytes in these jejunal fragments. As previously mentioned, GLP-2 increases intestinal blood flow and stimulates the expression of intestinal endothelial nitric oxide synthase (eNOS) (Guan et al., 2003). Inhibiting nitric oxide synthase with L-NAME does not impact intestinal-TRL secretion in hamsters (Hsieh et al., 2015), likely due to the lymphatic fate of these particles. Still, preventing GLP-2-mediated increases in portal and intestinal blood flow via L-NAME, blocks the GLP-2-mediated increase in apoB48 in the TRL fraction of plasma (Hsieh et al., 2015). Mice lacking endothelial nitric oxide synthase (*eNOS*^–/^*^–^* mice) display normal radiolabel appearance into plasma as wild-type controls, however, GLP-2 treatment in these mice did not increase plasma ^3^H compared to treatment in wild-type mice (Hsieh et al., 2015). ApoB48 in the TRL fraction is significantly lower in *eNOS^–/–^* mice compared to wild-type mice, independent of GLP-2 treatment (Hsieh et al., 2015). Additionally, jejunal TG mass is significantly greater in *eNOS^–/–^* mice compared to wild-type mice, again independent of GLP-2, suggesting that eNOS is involved in the release of stored TG as large chylomicrons rather than absorbed dietary TG and this is upregulated by exogenous GLP-2 (Hsieh et al., 2015). Indeed, GLP-2 treatment 5 h after 200 μL of intraduodenally administered olive oil significantly increases TRL-TG, which is inhibited by co-treatment with L-NAME. Similar to acute L-NAME treatment, L-NAME treatment alone 5 h post-oil does not change TRL-TG secretion compared to saline control (Hsieh et al., 2015), suggesting that GLP-2 may influence the partitioning of dietary fatty acids from lymph to portal circulation or that endogenous gut-hormone action by GLP-1R and/or GIPR maintains normal intestinal-TRL secretion. GLP-2 rapidly increases lymph flow and cumulative lymph volume in cannulated rats 300 min after an intraduodenal bolus of Intralipid 20% (Stahel et al., 2019). GLP-2 does not significantly change lymph TG concentration, TG output (mL TG per hour) or chylomicron size (TG:apoB48) compared to placebo control, rather it increases the cumulative increase in lymph TG in 60 min (Stahel et al., 2019).

### Regulation of Intestinal Growth and Response Injury

#### GLP-1R

Interestingly, *Gcgr^–/–^* mice have increased circulating GLP-1 and GLP-2 (Gelling et al., 2003; Ali et al., 2011; Grigoryan et al., 2012). Unsurprisingly, *Gcgr^–/–^* mice have significantly increased small and large intestinal length and weight due to the elevated levels of gut-derived hormones (Koehler et al., 2015). Consistent with this, co-administration of the GLP-2R agonist h(Gly^2^)GLP-2 and exendin-4 increases small intestinal weight and length to a greater extent compared to the agonists administered alone (Koehler et al., 2015). However, using *Gcgr^–/–^:Glp2r^–/–^* mice, the authors demonstrate that GLP-1R signaling can still increase small intestinal length and weight compared to wild type, although not to the same extent as in *Gcgr^–/–^* mice (Koehler et al., 2015). However, despite these potent effects, increased large bowel weight and length in *Gcgr^–/–^* mice appears to be driven by GLP-1R signaling as these parameters are unchanged in *Gcgr^–/–^:Glp2r^–/–^* mice (Koehler et al., 2015). Therefore, the trophic effects of GLP-1R signaling appear to target the distal gut (small intestinal length, large bowel length and weight) (Koehler et al., 2015). Indeed, exendin-4 and liraglutide treatment for 1 week increases small intestinal length and weight as well as large intestinal weight in wild-type mice but not in *Glp1r^–/–^* mice (Koehler et al., 2015). *Glp1r^–/–^* mice lose significantly more weight, exhibit significantly increased disease activity scores and greater colon damage than wild-type controls in response to DSS-induced colitis (Yusta et al., 2015). Unlike GLP-2, which increases crypt cell proliferation and villus elongation (Drucker and Yusta, 2014) exendin-4 treatment does not enhance crypt-cell proliferation, which was demonstrated by BrDU labeling and measuring crypt depth (Koehler et al., 2015). Instead, chronic treatment of exendin-4 increases crypt number in the proximal intestine and colon, leading to increased intestinal circumference and length (Koehler et al., 2015). The authors demonstrate that GLP-1R agonist treatment induces expression of tyrosine kinase IGF1R/ErbB (EGFR) pathways, however, agonist treatment can still increase intestinal growth in the absence of intestinal epithelial IGF1 receptor as well as EGF receptor signaling (Koehler et al., 2015). Acute, but not chronic, exendin-4 treatment increases *Fgf7* mRNA expression in the small intestine (Koehler et al., 2015). The intestinotrophic effects of exendin-4 are lost in *Fgf7^–/–^* mice, effects that were not observed upon GLP-2 treatment in these mice (Koehler et al., 2015). Despite the role of IELs in mediating intestinal mucosal repair through Fgf7/KGF (Boismenu and Havran, 1994), reconstituting *Glp1r+* IELs into *Glp1r^–/–^* mice via bone marrow transplant does not rescue the intestinotrophic effects of exendin-4 in these mice (Koehler et al., 2015).

Plasma GLP-1 levels increase in response to intestinal injury or mucosal inflammation (Zietek and Rath, 2016). IELs protect the epithelial barrier by promoting pathogen clearance and lysing pathogen-infected cells (Cheroutre et al., 2011). Treatment with exendin-4 significantly attenuates proinflammatory cytokines IL-2, IL-17a, interferon γ, tumor necrosis factor-α mRNA and protein in IELs activated by immobilized anti-CD3 and soluble anti-CD28, an effect partially blocked by GLP-1R antagonist exendin (9–39) (Yusta et al., 2015). Colonic mRNA expression analysis in *Glp1r^–/–^* mice at baseline reveal significant reductions in trefoil factor (*Tff-1* and -2), transforming growth factor (*Tgf-b1* and *Tgf-3*), epidermal growth factor receptor (*Egfr*), keratinocyte growth factor (*Fgf7*), hepatocyte growth factor (*Hgf*) (epithelial protection and repair), *Il6, Il1b* (innate immune response), *Il12b* (inflammation) (Yusta et al., 2015). Upon dextrane sulfate (DS)-induced colitis, colonic *Tff2, Tff3, Tgfb1*, and *Tgfb3* mRNA levels are significantly lower in *Glp1r^–/–^* mice compared to wild-type controls (Yusta et al., 2015). By contrast, colonic *Tgfb2* and *Ifng* mRNA levels are significantly higher in *Glp1r^–/–^* mice compared to wild-type controls (Yusta et al., 2015). Genes involved in innate immunity and inflammation, which are lower in *Glp1r^–/–^* at baseline, increases in both WT and *Glp1r^–/–^* in DSS-induced colitis, but differences between genotypes are lost (Yusta et al., 2015). Bone marrow transplantation, and therefore re-establishment of wild-type IELs in the intestinal mucosa, from wild-type donor mice to wild-type and *Glp1r^–/–^* recipient mice, normalizes colonic gene expression in response to DSS-induced colitis (Yusta et al., 2015). Exendin-4 increases mRNA expression of *Il1b, Il6, Il22, Il12b, Tnfa, Ccl2, Cxcl1*, and *Cxcl2* (innate immunity), regenerating islet-derived protein 2, *RegIIIy* and *RegIIIB* (anti-microbial proteins), as well as *Il-5, Il-13* (pathogen clearance) within 4 h of administration, returning to baseline expression by 24 h (Yusta et al., 2015), suggesting that GLP-1R activation engages a cytoprotective response. Exendin-4 treatment following DSS-induced colitis does not prevent weight loss, colon length shortening, or improve colon damage score, however, reductions in colon weight are attributable to a reduction of edema (Yusta et al., 2015).

#### GLP-2R

The intestinotrophic effects of GLP-2 have been well-described since its initial characterization (Drucker et al., 1997). GLP-2 increases intestinal cell proliferation while inhibiting apoptosis, leading to increased villus height and expanding the absorptive mucosal surface (Drucker et al., 1997). GLP-2 decreases mucosal injury by stimulating intestinal growth; specifically, increasing villus height, crypt depth, improving nutrient absorption and nutritional status (Estall and Drucker, 2005). Mice fasted for 24 h exhibit small intestinal atrophy, a decrease in intestinal weight, a decrease in crypt-villus height, and an increase in villus apoptosis (Shin et al., 2005). Refeeding restored all parameters, while co-administration of GLP-2R antagonist, GLP-2^3–33^, prevents adaptive changes to refeeding (Shin et al., 2005). Similarly, the restoration of jejunal mucosal mass, protein, and DNA 25–65% by *ad libitum* or intragastric infusion for 2–4 days is blunted with 2.5 or 50 μg/kg body weight GLP-2^3–33^, but not 10 μg/kg body weight GLP-2^3–33^, compared to the baseline fed group (Nelson et al., 2008). Mucosal growth following refeeding is associated with increased circulating GLP-2 and jejunal *Igf-1* mRNA expression. Interestingly, GLP-2^3–33^ at any dose prevents restoration of plasma IGF-I levels in response to refeeding (Nelson et al., 2008). There is evidence for both paracrine and neuronal mechanisms for GLP-2-mediated gut growth. GLP-2R+ myofibroblasts in the small intestine and colon contain keratinocyte growth factor (KGF), whereby immunoneutralization of KGF abolishes the trophic effects of GLP-2 treatment in the colon, but not the small intestine in mice (Orskov et al., 2005). Mechanistically, GLP-2 activates its receptors on subepithelial myofibroblasts, which in turn increase expression and secretion of IGF-1 (Dube et al., 2006). Gut growth coincides with increased IGF-1 and IGF-2, particularly in the mucosal and muscularis regions (Dube et al., 2006). GLP-2-mediated increases in IGF-1 activates the IGF-1 receptor on intestinal epithelial cells to stimulate proliferation (Rowland et al., 2011). Chronic GLP-2 treatment does not increase crypt-cell proliferation, and growth of the crypt-villus is reduced in intestinal epithelial-specific IGF knockout mice (Rowland et al., 2011).

Treatment of mice with DS-induced colitis (resembling human ulcerative colitis) with the human GLP-2 analog h(Gly^2^)GLP, twice daily for 10 days, reverses weight loss independent on food intake, decreases interleukin-1 expression and increased colon length, crypt depth, and mucosal area compared to treatment with saline (Drucker et al., 1999). h(Gly^2^)GLP-2 treatment also improves survival in drug-induced enteritis (non-steroidal anti-inflammatory drug - indomethacin) survival, reduces disease activity, decreases occurrence of intestinal ulcerations, and lowers cytokines and myeloperoxidase activity in mice (Boushey et al., 1999). In *Glp2r^–/–^* mice, levels of various Paneth cell genes are lower in the jejunum and ileum, some specifically in charge of defensin activity suggesting alterations in gut barrier function. The bacterial translocation and Paneth cell defect alter host-bacterial interactions within the intestine, further enhancing morbidity in *Glp2r^–/–^* mice (Lee et al., 2012). In the non-obese diabetic (NOD) mouse, a model of type 1 diabetes, treatment with h(Gly^2^)GLP-2 once daily for 14 days, increases small intestine length and weight, while also improving jejunal transepithelial resistance compared to treatment with saline. NOD mice treated with a single injection of h(Gly^2^)GLP-2 appear to have significantly decreased ion conductance in the jejunum (Hadjiyanni et al., 2009).

## Discussion

Agonism of GIPR, GLP-1R, and GLP-2R has a clear clinical impact on nutrient absorption and utilization; however, unraveling endogenous circuits’ location in mediating this beneficial effect has been challenging. Clearly, gut hormones represent a signal produced by cells in direct contact with nutrients, bacteria and circulation. Evaluation of models of metabolic disease and aging describe resistance to signaling through established GLP-1R+ circuits (Grasset et al., 2017; Varin et al., 2020). It is currently unclear if the resistance to GLP-1 is primarily due to impaired receptor expression, reduced signaling in the gut-brain axis and/or intestinal dysbiosis. It is also unknown how much of resistance of endogenous signaling contributes to the heterogeneity observed in metabolic disease and the variable patient responses to pharmacological treatments including DPP4 inhibitors, GLP-1R agonists and bariatric surgery.

As co-agonists are developed and proposed to have greater glycemic and intestinotrophic effects, further understanding of the endogenous signaling and target cells can only improve tailoring and outcomes.

## Author Contributions

NM, AH, and EM: writing—original draft preparation and review and editing. EM: funding acquisition. All authors have read and agreed to the published version of the manuscript.

## Conflict of Interest

The EM lab receives funding from the Merck IISP program for preclinical studies unrelated to this work. The remaining authors declare that the research was conducted in the absence of any commercial or financial relationships that could be construed as a potential conflict of interest.

## Publisher’s Note

All claims expressed in this article are solely those of the authors and do not necessarily represent those of their affiliated organizations, or those of the publisher, the editors and the reviewers. Any product that may be evaluated in this article, or claim that may be made by its manufacturer, is not guaranteed or endorsed by the publisher.

## Abbreviations

5-HT: serotonin receptor
ApoB48: apolipoprotein B48
ATP: adenosine triphosphate
AUC: area under curve
cAMP: cyclic adenosine monophosphate
ChAT: choline-acetyltransferase
CNS: central nervous system
DIRKO: double incretin receptor knockout
DPP4: dipeptidyl peptidase 4
DS: dextrane sulfate
EGFR: tyrosine kinase IGF1R/ErbB
eNOS: endothelial nitric oxide synthase
ER: endoplasmic reticulum
FABP5: fatty acid-binding protein 5
FFA: free fatty acid
FFAR1: free-fatty acid receptor 1
FFAR2: free-fatty acid receptor 2
FFAR4: free-fatty acid receptor 4
GAL_1_: galinin receptor
Gcg: preproglucagon
GCGR: glucagon receptor
GIP: glucose-dependent insulinotropic polypeptide
GIPR: glucose-dependent insulinotropic polypeptide receptor
GLP-1: glucagon-like peptide 1
GLP-1R: glucagon-like peptide 1 receptor
GLP-2: glucagon-like peptide 2
GLP-2R: glucagon-like peptide 2 receptor
GPCR: G-protein coupled receptor
GPR119: G-protein coupled receptor 119
GPR93: G-protein coupled receptor 93
GPRC6A: G-protein coupled receptor family C group 6 subtype A
IEL: intraepithelial lymphocyte
IL-1 β: interleukin 1 beta
IL-6: interleukin 6
KGF: keratinocyte growth factor
LCFA: long-chain fatty acid
LPL: lipoprotein lipase
LPS: lipopolysaccharide
nNOS: neuronal nitric oxide synthase
NOD: non-obese diabetic
OGTT: oral glucose tolerance test
PBS: phosphate-buffered saline
PC1/3: prohormone convertase 1/3
PC2: prohormone convertase 2
Pdx1: pancreatic and duodenal homeobox 1
Rfx6: regulatory factor X6
RYGB: Roux-en-Y gastric bypass
SCFA: short-chain fatty acid
SGLT1: sodium glucose co-transporter 1
SP: substance P
STAT: subtherapeutic antibody therapy
T2DM: type 2 diabetes mellitus
TG: triglyceride
TPN: total parenteral nutrition
TRL: triglyceride rich lipoprotein
VIP: vasoactive intestinal polypeptide
VSG: vertical sleeve gastrectomy
α SMA: smooth muscle actin.
